# Editorial: The Impact of Obesity on Cognitive Function

**DOI:** 10.3389/fnins.2022.916243

**Published:** 2022-06-15

**Authors:** Helen M. Melo, Natalia M. Lyra E Silva, Claudia A. Grillo

**Affiliations:** ^1^Institute of Medical Biochemistry Leopoldo de Meis, Federal University of Rio de Janeiro, Rio de Janeiro, Brazil; ^2^Centre for Neuroscience Studies, Department of Biomedical and Molecular Sciences, Queen's University, Kingston, ON, Canada; ^3^Department of Pharmacology, Physiology and Neuroscience, University of South Carolina School of Medicine, Columbia, SC, United States

**Keywords:** obesity, cognitive function, dementia, mental illness, brain

Obesity is a modern epidemic and a relevant public health concern worldwide. Multiple factors contribute to this global health burden, including genetic, behavioral, and social aspects. Lifestyle changes associated with our modern society led to an increased caloric intake, mainly sugars, trans and saturated fats, and reduced physical activity. Consequently, a chronic positive energy balance culminates in adipose tissue accumulation and pathological weight gain (Blüher, 2019). Besides the increased risk of non-communicable disease and premature death, being overweight and obese is associated with the development of cognitive decline and mental illness, including dementia (Singh-Manoux et al., 2018; Tang et al., 2021) and mood disorders (Fagiolini et al., 2003; Roberts et al., 2003; Herva et al., 2006; Bond et al., 2010). Considering the impact of cognitive dysfunction on quality of life, socialization, and occupational function, this Research Topic compiled a collection of articles investigating the effects of obesity on mental health.

The correct brain structure and connectivity are critical components in the coordination and regulation of psychological and behavioral responses related to food evaluation, mainly in regions involved in reward, emotion, and cognition. To understand how alterations in neural structure and hierarchical activity are associated with obesity, Zhang et al. recruited normal-weight and obese subjects to investigate structural changes and neurocircuit reorganization of the brain. The analysis of functional images from forty-two participants with obesity (BMI > 30 kg/m^2^) and thirty-three normal-weight volunteers (18.5 kg/m^2^ < BMI <25.0 kg/m^2^) (18–55 years old) showed increased gray matter volume (GMV) alterations in the parahippocampal gyrus (PG) in the obese group. Moreover, when analyzed at the effective connectivity level, obesity was related to decreased interactions between the PG and orbitofrontal cortex (OFC) and the PG and supplementary motor area (SMA). Additional results also indicate alterations in causal outflow and DDEBQ-External/Restrain scores between PG and other brain regions. Despite some limitations related to sample size and the control of other factors that would affect the whole-brain activity, this study highlights the relevance of brain networks, mainly between regions involved in hedonic and motivation in the obese pathology.

One of the consequences of weight gain is the excessive accumulation of visceral fat and the establishment of central or abdominal obesity, which has a pivotal role in metabolic syndrome (MeS) development. MeS, a health condition that combines diabetes, hypertension, and obesity, increases the risk of cardiovascular diseases, stroke, and other disorders (Després and Lemieux, 2006). Interestingly, MeS is more prevalent in patients with bipolar disorder (BD) (Fagiolini et al., 2005; Vancampfort et al., 2013). Thus, to explore the mechanism underlying cognitive decline in subjects with BD and MeS comorbid, Dalkner et al. evaluated neuropsychological aspects including attention and processing speed, verbal memory, and executive function in healthy controls (HC) and BD subjects (aged between 18 and 70 years; 52,5% female). Notably, subjects with MeS and BD comorbid showed impaired executive function comparing BD subjects without MeS and HC with or without MeS. The study did not find significant interactions between MetS and BD concerning attention/processing speed and verbal learning/memory. These results highlighted the relevance of considering the MeS risk in the clinical management of BD, mainly in cognitive aspects of the disease.

Due to the complexity and multifactorial aspect of obesity pathogenesis, many studies have investigated different biochemical and cellular mechanisms involved in obesity-induced cognitive dysfunction. Here, Olsthoorn et al. review key studies published in the past decade showing the contribution of inflammatory signaling and the interconnection of microbiota changes, white adipose tissue (WAT) accumulation, and increased blood-brain barrier (BBB) permeability to a neuroinflammatory response and alteration in the brain's structure that underlies the cognitive decline in obesity.

Another issue in understanding the effects of obesity on the brain has been the role of dietary patterns. Although the study of diet components in humans still needs to be further investigated, evidence points to the action of nutritional content on brain health. In this line, the Mediterranean diet, enriched with fruit, vegetables, fish, and whole grains, was associated with a reduced risk for depression and cognitive impairment (Psaltopoulou et al., 2013). On the other hand, the Western diet, enriched in ultra-processed foods, processed and red meat, high-sugar drinks, and fried foods, was associated with dementia and psychiatric diseases (Kalmijn et al., 1997; Jacka et al., 2010; Barnard et al., 2014). Additionally, brain diseases, including dementia and mental illnesses, affect men and women differently (Seedat et al., 2009; Beam et al., 2018), and it remains to be understood possible sex differences associated with diet effects (Brennan and Gibbons, 2020). In accord with this, Huq et al. reported a potential function of sex and brain serotonin (5-HT) in molecular signaling and behavior in mice chronically fed with high-fat diet (HFD). The authors compared the effects observed in a previous study using a tryptophan hydroxylase 2 (R439H) knock-in male mice, a genetic model of brain serotonin (5-HT) deficiency, with the present study using female (R439H) knock-in mice. Regardless of the statistical limitation in comparing male and female models due to time between experiments, the authors reported a relevant sex-dependent effect on weight gain, anxiety, and depressive behavioral tasks. Correspondingly, the HFD effects on hippocampal molecular signaling differ according to sex. Female mice are less sensitive to weight gain and showed a different neuroinflammatory pattern and GSK3β signaling in the hippocampus. Overall, the results indicate a potential role of 5-HT signaling in hippocampal-dependent behavior response to chronic HFD consumption in a sex-dependent manner.

Finally, de Paula et al. investigated the effects of short-term HFD consumption on BBB permeability and neuroinflammation in mice. Intriguingly, HFD showed a rapid impact on mice behavior. Three and five days after the HFD, mice exhibited memory impairment and depressive-like behavior, respectively. Importantly, before behavioral changes (1 and 2 days after HFD exposure), mice showed increased BBB permeability in the hippocampus. In the same way, this brain region revealed a transient increase in TNF-α and IL-6 mRNA. Besides, increased astrocytes activation, reduced synaptic density, and mitochondrial dysfunction occurred later. These results reinforce the importance of understanding the role of diet components in brain health.

Overall, this Research Topic might provide new insights into the brain alterations underlying obesity-induced cognitive decline. Hence, we aspire to increase the understanding of how obesity and its co-morbidities affect brain health to establish preventive strategies that improve the human quality of life.

## Author Contributions

HM drafted this editorial. NL and CG each contributed by revising and editing. All authors listed contributed to the work and approved it for publication.

## Conflict of Interest

The authors declare that the research was conducted in the absence of any commercial or financial relationships that could be construed as a potential conflict of interest.

## Publisher's Note

All claims expressed in this article are solely those of the authors and do not necessarily represent those of their affiliated organizations, or those of the publisher, the editors and the reviewers. Any product that may be evaluated in this article, or claim that may be made by its manufacturer, is not guaranteed or endorsed by the publisher.
